# Identification of the Reference Genes for Relative qRT-PCR Assay in Two Experimental Models of Rabbit and Horse Subcutaneous ASCs

**DOI:** 10.3390/ijms25042292

**Published:** 2024-02-14

**Authors:** Zhenya Ivanova, Valeria Petrova, Natalia Grigorova, Ekaterina Vachkova

**Affiliations:** Department of Pharmacology, Animal Physiology, Biochemistry and Chemistry, Faculty of Veterinary Medicine, Trakia University, 6000 Stara Zagora, Bulgaria; valeria.petrova1075@gmail.com (V.P.); n.gr@abv.bg (N.G.); katvach@gbg.bg (E.V.)

**Keywords:** glyceraldehyde 3-phosphate dehydrogenase (*GAPDH*), hypoxanthine-guanine phosphoribosyltransferase (*HPRT*), endogenous control, adipose tissue, equine, rabbit

## Abstract

Obtaining accurate and reliable gene expression results in real-time RT-PCR (qRT-PCR) data analysis requires appropriate normalization by carefully selected reference genes, either a single or a combination of multiple housekeeping genes (HKGs). The optimal reference gene/s for normalization should demonstrate stable expression across varying conditions to diminish potential influences on the results. Despite the extensive database available, research data are lacking regarding the most appropriate HKGs for qRT-PCR data analysis in rabbit and horse adipose-derived stem cells (ASCs). Therefore, in our study, we comprehensively assessed and compared the suitability of some widely used HKGs, employing RefFinder and NormFinder, two extensively acknowledged algorithms for robust data interpretation. The rabbit and horse ASCs were obtained from subcutaneous stromal vascular fraction. ASCs were induced into tri-lineage differentiation, followed by the eicosapentaenoic acid (EPA) and docosahexaenoic acid (DHA) treatment of the adipose-differentiated rabbit ASCs, while horse experimental groups were formed based on adipogenic, osteogenic, and chondrogenic differentiation. At the end of the experiment, the total mRNA was obtained and used for the gene expression evaluation of the observed factors. According to our findings, glyceraldehyde 3-phosphate dehydrogenase was identified as the most appropriate endogenous control gene for rabbit ASCs, while hypoxanthine phosphoribosyltransferase was deemed most suitable for horse ASCs. The obtained results underscore that these housekeeping genes exhibit robust stability across diverse experimental conditions, remaining unaltered by the treatments. In conclusion, the current research can serve as a valuable baseline reference for experiments evaluating gene expression in rabbit and horse ASCs. It highlights the critical consideration of housekeeping gene abundance and stability in qPCR experiments, emphasizing the need for an individualized approach tailored to the specific requirements of the study.

## 1. Introduction

Polymerase chain reaction (PCR) is an exceptionally preferred and dependable technique for scrutinizing gene expression, and real-time PCR stands as a favored approach due to its remarkable sensitivity, susceptibility, and reproducibility [1]. However, some factors can easily affect the method’s accuracy and precision. The reliability of the obtained results largely depends on the quality and quantity of mRNA used as a template for the qRT-PCR reaction, as well as on factors such as the efficiency of the reverse transcription reaction, primer design and concentration, incubation time, running temperature, annealing temperature, template concentration, and reaction conditions [2,3]. Nevertheless, even if all steps are performed correctly, a crucial aspect that can affect the analysis results’ accuracy still remains. To mitigate the effect of changes in experimental conditions, including media composition, cell morphological variations, and other influencing qRT-PCR analysis factors, selecting an appropriate internal standard or reference gene, commonly referred to as a housekeeping gene, is imperative. The optimal reference gene is characterized by stable expression levels without being significantly affected by metabolic changes or exposure to diverse substances. Regardless of the cell differentiation stage, its expression remains constant and comparable to the target gene across different tissue types [4]. Ideally, the internal control gene should be associated with vital cellular processes, such as primary metabolism or cellular structure [5,6].

Several web-based analysis tools, such as NormFinder [7], BestKeeper [8], geNorm [9], and RefFinder [10], have been developed to evaluate the stability and suitability of candidate reference genes for real-time PCR data normalization in a given set of biological samples. However, they significantly differ in their approach, and the results obtained by the distinct mathematical algorithms can be quite contradictory [11].

It should also be emphasized that utilizing universal reference genes across diverse sample types and experimental conditions is not feasible [12]. Traditional housekeeping genes commonly employed for normalization, such as hypoxanthine phosphoribosyl transferase 1 (*HPRT1*), *18S*, β-actin (*ACTB*), and glyceraldehyde-3-phosphate dehydrogenase (*GAPDH*), often exhibit substantial variability in expression due to their multifaceted and context-dependent functionality [5,13,14].

Previously, *GAPDH* was commonly considered a stably expressed gene primarily associated with glycolysis, making it a viable candidate for internal control in various experimental studies. However, recent research reveals that *GAPDH* has a vital role in a variety of other cellular processes, including DNA repair [15] and tRNA export, by regulating the components of the nuclear pore complex, involved in the transport of molecules between the cytoplasm and the nucleus [16]. *GAPDH* has been implicated in the fusion of vesicles with cell membranes and the regulation of cytoskeletal components, contributing to cell structure, movement, and division [17]. Finally, *GAPDH* has been linked to cell death as a mediator of programmed cell death (apoptosis) and has been considered a potential factor in cell death associated with neurodegenerative diseases [18]. These findings suggest that *GAPDH* has a much broader range of cell functions than was initially recognized and may be a key player in various cellular processes beyond glycolysis [19].

Another widespread housekeeping gene is *HPRT*, encoding an enzyme engaged in the purine salvage pathway, which recycles purine bases such as hypoxanthine and guanine to produce nucleotides for DNA and RNA synthesis [20]. *HPRT* catalyzes the transfer of a phosphoribose group from phosphoribosyl pyrophosphate to either hypoxanthine or guanine, producing inosine monophosphate or guanosine monophosphate, respectively. These nucleotides can be further converted to other nucleotides, such as adenosine monophosphate and cytidine monophosphate, or incorporated directly into nucleic acids. Since guanosine triphosphate is required for both DNA synthesis and as an energy molecule in the cell, *HPRT* is considered a housekeeping gene produced at relatively constant levels in all somatic tissues [21]. Its stable expression makes it a commonly used endogenous control or reference gene in gene expression studies [22]. However, its stability during metabolically active states, such as stem cell differentiation, warrants further investigation.

Similarly, β-actin and *18S* are commonly used as reference genes because they are permanently expressed in most eukaryotic cells and are considered relatively stable under different experimental conditions [23]. As a cytoskeletal protein, β-actin is involved in cell structure and movement, and its mRNA is transcribed continuously in most cells. As a component of the small ribosomal subunit, 18S rRNA is required for protein translation [24].

However, it is imperative to highlight that the stability and appropriateness of *GAPDH*, *HPRT*, *18S*, and β-actin as reference genes are subject to the influence of diverse factors, including sample type, experimental treatments, and normalization methods, especially when cells undergo functional morphological alterations [25,26,27]. Therefore, it is crucial to validate the expression stability of candidate reference genes under specific trial conditions before using them in gene expression analyses.

Adipose-derived stem cells (ASCs) have emerged as valuable human and animal research model systems. The utilization of ASCs provides numerous advantages in experimental investigations. Firstly, adipose tissue is an easily accessible and abundant source of stem cells, mitigating ethical concerns and simplifying experimental procedures. Furthermore, ASCs can be readily isolated and expanded in culture, facilitating large-scale experiments and allowing for repeated sampling. The remarkable differentiating capacity of ASCs across multiple cell lineages further enables studying diverse biological processes and disease models [28,29,30].

A notable trend has emerged in recent years wherein traditional cell-based experimental models employing mice and rats are substituted with stem cells isolated from rabbits and horses. This shift is particularly evident in research on regenerative medicine, cellular therapies, obesity, lipolytic nutritional supplements, and diabetes [31,32]. Horses are widely acknowledged and well-established animal models in regenerative medicine, especially for musculoskeletal disorders, due to their clinical relevance and firmly established nature [33,34]. Several diseases affecting animals and humans, including those related to development, infection, autoimmunity, or allergies, share a common underlying physiological basis. Therefore, using animals like horses as test models to evaluate the therapies’ effectiveness and safety in human medicine appears to be a reasonable approach [35]. On the other hand, rabbits seem very promising, as they have lipid metabolism and a lipid profile similar to humans, which makes them suitable for testing the influence of different additives, such as omega-3 polyunsaturated fatty acids (PUFAs), on obesity markers [36]. However, to our knowledge, an evaluation of suitable reference genes for normalizing gene expression in equine and rabbit ASCs has received limited attention.

The aim of this study was to identify the most suitable endogenous control gene for normalizing qRT-PCR data in adipose-derived stem cells from horses and rabbits. We evaluated the stability of expression of widely accepted reference genes (*18S*, *GAPDH*, and *HPRT*), in adipogenically induced rabbit ASCs treated with frequently used omega-3 PUFAs (eicosapentaenoic (EPA) and docosahexaenoic acid (DHA)) and in equine ASCs undergoing adipogenic, chondrogenic, and osteogenic differentiation. A key strength of our research is its contribution to filling a gap in the scientific field by validating the most suitable gene in ASCs within the chosen experimental conditions. Unlike other studies, in which researchers often rely solely on literature data when selecting a control gene, this research provides prevalidation through the experimental testing of these commonly used housekeeping genes.

## 2. Results

### 2.1. Identification and Characterization of ASCs

**Figure 1:**
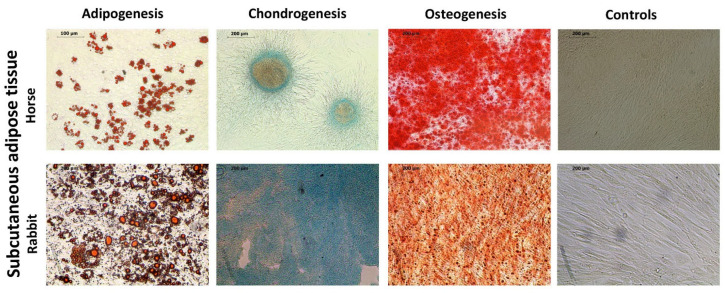
Tri-lineage differentiation of horse and rabbit ASCs: for adipogenesis, the samples were stained with Oil Red O, scale bars 100 μm (horses) and 200 μm (rabbits); for chondrogenesis, the samples stained with Alcian Blue, scale bars 200 μm; for osteogenesis, the samples were stained with Alizarin Red, scale bars 200 μm; controls, scale bars 200 μm.

Tri-lineage differentiation was performed to identify and characterize ASCs (adipogenic, chondrogenic, and osteogenic) (Figure 1). The results of histochemical staining demonstrate typical cell inclusions, such as intracellular lipid droplets, extracellular cartilage-specific proteoglycans, and calcium deposits.

### 2.2. Expression Stability Evaluation of the Reference Genes Using Two Popular Algorithms

We analyzed three distinct experimental groups of rabbit ASCs that previously underwent adipogenic differentiation. Two of the three groups were treated with EPA and DHA, while the third group served as the untreated control. In the equine analyses, we assessed the gene stability of three groups categorized according to differentiation type: adipogenic ASCs (aASCs), osteogenic ASCs (oASCs), and chondrogenic ASCs (chASCs).

#### 2.2.1. NormFinder

NormFinder ranks the set of candidate internal control genes based on their expression stability using inter- and intragroup variations. The most stable reference genes within and between the groups are those with the lowest stability value [7]. According to this approach, in rabbit subcutaneous adipose tissue, the best housekeeping gene was *GAPDH*, while in horse ASCs, *HPRT* was the most stable reference gene (Table 1).

#### 2.2.2. RefFinder

**Figure 2:**
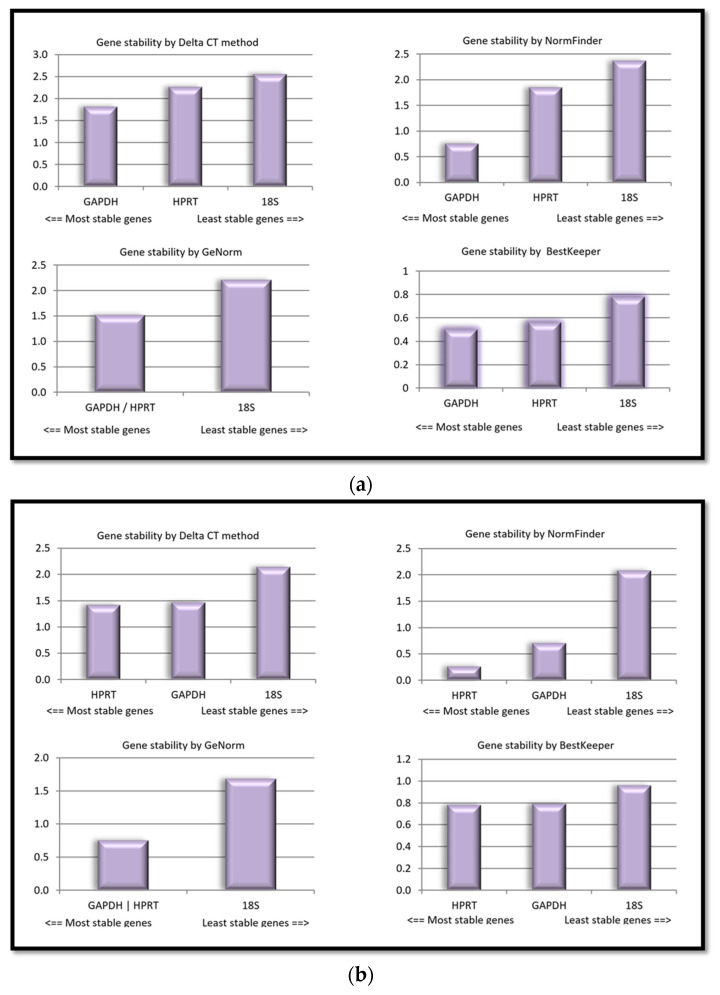
The stability ranking of candidate housekeeping gene identified by RefFinder in rabbit and horse ASCs (**a**,**b**), respectively, based on their Ct values. The following groups were analyzed: for rabbit ASCs, control as well as EPA- and DHA-treated groups; for horse ASCs, the groups undergoing adipogenic, osteogenic, and chondrogenic differentiation.

RefFinder, a web-based software program, integrates different computational algorithms, including geNorm, NormFinder, and BestKeeper, to evaluate the stability of candidate normalization genes and rank them accordingly [10]. RefFinder uses the comparative delta Ct method to compare the expression levels of different candidate reference genes. It is important to note that the stability of reference genes may vary depending on the specific experimental conditions, and it is recommended to validate the selected reference genes for each experiment. The use of multiple algorithms and integrated computational programs, as provided by RefFinder, can help enhance the accuracy and reliability of reference gene selection in gene expression studies. Figure 2a,b depict the expression stability ranking of the three candidate reference genes in rabbit and horse ASCs.

The results indicate that significant biological differences between the two species contribute to the observed gene expression variations. Based on the data from the experiment, it was affirmed with a high degree of confidence that *GAPDH* had the most exceptional stability among the tested genes in rabbit ASCs. However, in horse ASCs, *HPRT* was confirmed to exhibit the highest level of stability.

### 2.3. Expression Levels and Variation in Candidate Housekeeping Genes

An inverse correlation exists between the Ct value and gene expression levels. A lower Ct value signifies higher gene expression, while a higher Ct value indicates lower gene expression [6]. Nevertheless, genes with a standard deviation (SD) value exceeding one are considered unstable, aligning with our findings (Table 2).

### 2.4. Efficiency of the Reactions

PCR efficiency refers to how effectively the target DNA is amplified with each reaction cycle. According to the obtained results from PCR reactions, the efficiency values of examined housekeeping genes varied between 95% and 100%, underscoring their accuracy and reliability in the amplification process (Table 3).

The slope of the standard curve is indicative of the amplification efficiency of the PCR reaction. An efficiency of 100% corresponds to a slope of −3.32 in the standard curve. Deviations from this slope indicate variations in the amplification efficiency of the reaction.

## 3. Discussion

Stem cells can regenerate the functionally and anatomically impaired integrity of an organ due to their ability to differentiate into various classes of specialized cell types [37]. Among these, mesenchymal stem cells (MSCs) are widely regarded as promising in therapeutic interventions owing to their ease of isolation, storage, cultivation, differentiation, and secretion of regenerative bioactive factors. Historically, the prevailing belief was that MSCs predominantly originated from bone marrow. However, contemporary research has outlined adipose tissue as a preferred and even more abundant source of these cells, offering facilitated accessibility and less invasive harvesting methods [38]. Consequently, adipose-derived stem cells (ASCs) have become increasingly prominent in both human and animal therapies and serve as suitable experimental models in research areas like obesity, cancer, tissue engineering, and drug discovery [39,40,41]. In molecular research on these stem cells, a critical aspect is the selection of appropriate reference genes for gene expression analysis. During differentiation, ASCs undergo significant morphological and physiological changes, complicating the identification of a stable and suitable internal control gene, a challenge further deepened by variability across various biological species and experimental conditions.

Our study was focused on identifying a suitable internal control gene in ASCs from subcutaneous adipose tissue from two poorly investigated animal species—horses and rabbits—during tri-lineage differentiation—adipogenesis, osteogenesis, and chondrogenesis. According to the obtained results, *GAPDH* exhibited the most stable expression in adipogenically differentiated ASCs isolated from rabbits and treated with omega-3 PUFAs, while *18S* and *HPRT* showed significant variability. *GAPDH* is extensively employed as a housekeeping gene and is widely preferred in genomic studies, primarily due to its pivotal role in glycolysis—a central metabolic pathway crucial for cellular energy production. Its expression remains consistent across various experimental conditions, cell types, and developmental stages [42,43,44]. However, its suitability in the context of adipogenesis is still questionable due to *GAPDH’s* involvement in triglyceride synthesis. Some researchers consider *GAPDH* as a later marker of adipogenesis and believe that its expression reflects its dynamic role in the lipid accumulation phase of adipocyte development [45,46,47]. By contrast, Gorzelniak et al. [48] identified *GAPDH* as a housekeeping gene with the highest stability both in preadipocytes and mature adipocytes, regardless of hormone exposure. In the context of adipogenesis, other researchers have also reported that *GAPDH* exhibits the most stable expression and, similar to our results, outlined *18S* as an unreliable internal control [49,50].

Over the past few years, numerous scientific studies have focused on different research fields related to equine ASCs. However, a notable gap exists in the literature concerning the validation of internal controls for ASCs. Investigations on the stability of housekeeping genes in adipose tissue across different species, including horses, humans, rats, and rabbits, have highlighted genes such as *18S*, *ACTB*, *GAPDH*, and *HPRT* for their consistent expression [51,52,53]. In equine experiments, many researchers utilize housekeeping genes like *HPRT* as internal control without directly verifying its stability [54,55,56]. Bruynsteen et al. [57] identified both *HPRT* and *GAPDH* as the most stable housekeeping genes in equine adipose tissue. Contrarily, some authors have shown significant variability in *GAPDH* gene expression under certain treatment conditions [27,58,59].

Our research identified *HPRT* as a stable housekeeping gene in horse ASCs undergoing tri-linear differentiation, namely adipogenesis, osteogenesis, and chondrogenesis, suggesting its potential utility under these specific conditions. Almeida-Oliveira et al. [27] highlighted that selecting suitable housekeeping genes for research is notably influenced by the validation methodology employed. Their study revealed that using multiple software tools for validation often leads to conflicting results, further complicating the identification of a reliable internal control for experimental studies. The research methodology in our experiment includes two software programs—NormFinder and RefFinder. NormFinder, the most commonly used tool, is based on a mathematical model ranking candidate normalization genes on their expression stability [7]. It assesses the gene variation within and between sample groups for proper estimation across the experimental conditions by calculating the coefficient of variation (CV) and pairwise variation (V). Genes with lower CV or V values are considered more stable and are prioritized for selection as reference genes. According to the software analysis, *HPRT* showed less intra- and intergroup variations in horse ASCs, while in rabbit ASCs, *GAPDH* was the most unvarying. To confirm the results obtained from NormFinder, we further evaluated the raw Cq values using RefFinder, which has recently emerged as a comprehensive alternative tool that amalgamates various commonly used algorithms—NormFinder, BestKeeper, delta Ct, and geNorm. BestKeeper, an Excel-based tool, interprets the data based on the variations in Ct values and intergene relations in all reference pairs (Pearson correlation). Genes exhibiting a standard deviation (SD) value exceeding one are regarded as unstable (A). The delta Ct method compares the relative expression of pairs of housekeeping genes within each sample and progressively eliminates these with larger delta Ct value deviations (B). The geNorm algorithm calculates the average pairwise variation between a reference gene and all other genes, resulting in an M value. The lowest M value signifies the gene with the highest degree of stability (C). Finally, RefFinder generates a summarized report based on the coefficient of variation (CV) and pairwise variation (V) and provides a more robust evaluation of gene stability compared to single-measure tools. Its iterative refinement process enhances the accuracy of results, making it suitable for complex datasets [10].

De Spiegelaere [60] claimed that the implementation of the original software packages (BestKeeper, NormFinder, and geNorm) generated quite different outcomes compared to the results obtained by RefFinder. The most probable reason is the lack of consideration for PCR efficiencies. When the raw data were reanalyzed, assuming 100% efficiency for all genes, the outputs of the original software packages closely resembled those generated by the RefFinder software.

## 4. Materials and Methods

Before performing the collection, horses were sedated intravenously with a mixture of 0.01 mg/kg of detomidine hydrochloride (Domosedan, Orion Pharma, Finland) and 0.025 mg/kg of butorphanol tartrate (Butomidor, Richter Pharma, Austria), followed by local infiltration with 5 mL of lidocaine 2% solution (Sopharma, Bulgaria) for regional desensitization. Additionally, rabbits were sacrificed based on the recommendations and approval of the Local Committee for Human Treatment of Animals. Adipose tissue samples weighing 1–1.5 g were then obtained from the gluteal region subcutaneous layer of 18-year-old mix breed horses through a longitudinal skin incision in the Regio radical caudal [61], while in rabbits, the samples were obtained from the subcutaneous (interscapular region) depots of 28-day-old New Zealand rabbits. The isolation procedure was performed according to Vachkova et al. [62]. Initially, the samples were minced in a laminar flow hood, collagenase-digested for 2 h, and centrifuged at 300× *g* for 10 min. The resulting pellet representing a heterogeneous population of cells known as a stromal vascular fraction (SVF) was resuspended and seeded into T25 tissue flasks in cell culture media containing Dulbecco’s modified Eagle’s medium (DMEM), 10% fetal bovine serum (FBS), and 10 mL/L antibiotic–antimycotic solution, all from Sigma (St. Louis, MO, USA). The cells were cultured and incubated under humidified, 5% CO_2_ conditions at 37 °C until reaching confluence.

### 4.1. Experimental Design Rabbits

The cells were cultured up to passage four and further induced in an adipogenic differentiation for 21 days. At this stage, the following groups were formed: control group, treated with DMEM; FBS, L-glutamine, antibiotic mixed solution (control medium); EPA group, treated with the control medium supplemented with 100 µg EPA; and DHA group, treated with the control medium + 100 µg DHA. The cells were cultured in these conditions for 48 h. The cells were additionally induced in osteogenic and chondrogenic differentiation media to demonstrate their functional multipotency. The protocols of tri-lineage differentiation were performed as described by Vachkova et al. [62].

### 4.2. Experimental Design Horses

The functional multipotency of the isolated cells was assessed through adipogenic (aASC group), osteogenic (oASC group), and chondrogenic (chASC group) differentiation. The fifth passage cells were seeded in 12-well plates at a density of 5 × 10^5^/mL in a basal medium with FBS until reaching confluence.

#### 4.2.1. Adipogenic Differentiation

The cells were induced in an adipogenic medium (DMEM-HG, 10% HS (heat-inactivated horse serum), 1 μM dexamethasone, 10 μg/mL insulin, 0.05 mM indomethacin, 0.5 mM IBMX, and 10 mL/L antibiotic–antimycotic solution) for 18 days. To visualize the intracellular lipid droplets (LDs), the cells were stained with Oil Red O.

#### 4.2.2. Osteogenic Differentiation

The cells were treated with an osteogenic medium (4% HS, 1% ITS, 0.1 μM dexamethasone, 10 mM β-glycerolphosphate, 50 µM L-ascorbic acid, 20 mL/L L-glutamine, 10 mL/L antibiotic–antimycotic solution, and DMEM, all from Sigma). After 21 days, Alizarin Red S was applied to the cells to observe the calcium deposits outside the cells.

#### 4.2.3. Chondrogenic Differentiation

The cells were cultured with chondrogenesis-inducing media (1% ITS, 4% HS, 0.1 μM dexamethasone, 10 ng/mL TGFβ1, 50 µM L-ascorbic acid, 20 mL/L L-glutamine, 10 mL/L antibiotic–antimycotic solution, and DMEM, all from Sigma) for 21 days. The visualization of extracellular cartilage-specific proteoglycans was carried out by utilizing Alcian Blue staining.

#### 4.2.4. Negative Controls

Non-induced ASCs were cultured in parallel only with the basic growth medium and used as negative controls for each type of differentiation, stained with Oil Red O, Alizarin Red S, and Alcian Blue, respectively.

Stained cells were subsequently observed under a Leica DM1000 LED inverted microscope (Heerbrugg, Switzerland), equipped with a 5.0 MP resolution DMi1 camera version and the software platform Leica Application Suite Core, used for all micrographs.

### 4.3. Gene Expression Analysis (RT-qPCR)

#### 4.3.1. Rabbit Protocol

For the isolation and purification of the total RNA from subcutaneous tissue samples, an RNeasy Lipid Tissue Kit from Qiagen (Hilden, Germany) was used. Then, to determine the RNA quantity, the absorbance was measured at 260 and 280 nm with a GeneQuant 1300 spectrophotometer (GE Healthcare Bio-Sciences AB, Uppsala, Sweden). Reverse transcription reaction was implemented with a RevertAid First Strand cDNA Synthesis Kit (Fermentas Life Science, Thermo Fisher Scientific™, Waltham, MA, USA) using a Quanta Biotech QB-96 cycler (Quanta Biotech Ltd., Surrey, UK). cDNA was stored at −20 until use. The real-time PCR reaction was carried out using the ABI Prism 7900H sequence detection system (AppliedBiosystems, Foster City, CA, USA).

#### 4.3.2. Horse Protocol

The total cellular RNA was extracted using a Universal RNA Purification Kit (EURx, Gdańsk, Poland) per the manufacturer’s protocol. BioTek’s Take3 plate measured RNA purity and quantity, Synergy™ LX Multi-Mode Microplate Reader (BioTek, Santa Clara, CA, USA) at 260 and 280 nm absorbance. All RNA samples were purified from genomic DNA using RNAse-Free DNase Set (Qiagen, Germany) and were reverse-transcribed using a RevertAid First Strand cDNA Synthesis Kit (Thermo Scientific, Waltham, MA, USA), following the manufacturer’s instructions. The analysis of the expression levels of the housekeeping genes, *18S*, *HPRT*, and *GAPDH*, was performed with real-time qPCR thermocycler Gentier 96E (Xi’an Tialong Science and Technology, Xi’an, China) using KAPA SYBR FAST Master Mix (2X) (Roche) in duplicates with two-step qPCR. The amplification of cDNA was performed following the manufacturer’s conditions.

#### 4.3.3. Standard Curve Construction

The efficiency of the PCR reaction was assessed by generating a standard curve. This curve was constructed using a five-fold serial dilution of a pool of all the DNA samples used in the PCR reaction following the above-mentioned algorithm. Finally, PCR software (Xi’an Tianlong Science and Technology Co., Ltd., https://www.medtl.net/ accessed on 19 February 2023) automatically calculated the efficiency, based on the slope of the standard curve.

The primers for all housekeeping genes were designed using open access data in NCBI (www.ncbi.nlm.nih.gov, accessed on 19 February 2023) and Ensembl (www.ensambl.org, accessed on 22 February 2023). The product length and annealing temperatures were determined via the web-based software Primer 3 (http://bioinfo.ut.ee/primer3/, accessed on 25 February 2023). The primer sequences for the housekeeping genes are presented in Table 4. The primer sequence of *18S* for horse samples was designed by Arnhold et al. [63].

### 4.4. Evaluation of Expression Stability of Housekeeping Genes

We employed two commonly used algorithms to identify the most stable and suitable endogenous control: NormFinder (an Excel add-in, MS Office version 2016) [7] and RefFinder (http://blooge.cn//RefFinder/, accessed on 25 February 2023) [10].

### 4.5. Statistical Analysis

To evaluate the standard error of the mean (SEM) and the mean values for each gene, Statistica v.10 (StatSoft, Inc., Tulsa, OK, USA (2011) was used. Furthermore, the least significant digit (LSD) test was performed to estimate intergroup variations. A statistically significant difference was considered as a *p*-value of less than 0.05.

## 5. Conclusions

In conclusion, our investigation demonstrated that *GAPDH* exhibited stable expression in rabbit ASCs after adipogenesis and treated with omega-3 PUFAs. At the same time, *HPRT* was identified as a stable internal control in horse ASCs during tri-lineage differentiation. This research emphasizes the importance of a precise validation methodology, employing multiple software tools such as NormFinder and RefFinder, to comprehensively assess gene stability. The choice of appropriate housekeeping genes should be tailored to the specific requirements of each trial, highlighting the need for an individual approach.

We believe that our findings can contribute to selecting suitable internal controls in ASCs, particularly in less-explored species, and highlight the significance of methodological integrity in gene expression studies involving stem cells.

## Figures and Tables

**Table 1:** Expression stability evaluation of the reference genes using NormFinder, based on their quantity.

Gene Name	Stability Value	
	Rabbit	Horse
*18S*	0.473	0.589
*GAPDH*	**0.136**	0.235
*HPRT*	0.263	**0.071**

**Table 2:** Mean threshold cycles (Ct) and standard deviation (SD) of *18S*, *GAPDH*, and *HPRT* in rabbit and horse ASCs.

Gene Name	Mean Ct Values with Their SD	
	Rabbit	Horse
*18S*	17.01 ± 1.05	19.03 ± 1.15
*GAPDH*	23.28 ± 0.40	23.48 ± 1.26
*HPRT*	27.74 ± 0.88	26.91 ± 0.94

**Table 3:** Slope and efficiency determination by standard curve analysis in qPCR of *18S*, *GAPDH*, and *HPRT* genes in rabbit and horse ASCs.

Gene Name	Slopes/Efficiency (%) of PCR Reaction	
	Rabbit	Horse
*18S*	−3.449/94.96	−3.389/97.28
*GAPDH*	−3.333/99.54	−3.337/99.38
*HPRT*	−3.331/99.62	−3.345/99.04

**Table 4:** Primer sequences, product length, and accession number of candidate reference genes for qPCR normalization in adipose-derived stem cells.

Abbreviation	Full Name	Forward	Reverse	Product Length	NCBI Accession №
**HORSE**					
***HPRT***	Hypoxanthine phosphoribosyltransferase	CCAGTCAACAGGGGACATAA	GCTTGCGACCTTGACCATCT	**163**	AY372182.1
***GAPDH***	Glyceraldehyde 3-phosphate dehydrogenase	TCCCTGCTTCTACTGGTGCT	CGTATTTGGCAGCTTTCTCC	**147**	NM_001163856.1
***18S***	18S ribosomal RNA	ATGCGGCGGCGTTATTCC	GCTATCAATCTGTCAATCCTGTCC	**204**	NR_046271.1
**RABBITS**					
***HPRT***	Hypoxanthine phosphoribosyltransferase	CCCCAGCGTTGTGATTAGTG	GCCTCCCATCTCCTTCATCA	**163**	ENSOCUT00000030995.2
***GAPDH***	Glyceraldehyde 3-phosphate dehydrogenase	GAACGGGAAGCTGGTCATCA	GAAGACGCCAGTGGATTCCA	**118**	GCA_000003625.1
***18S***	18S ribosomal RNA	ATCAGATACCGTCGTAGTTC	TTCCGTCAATTCCTTTAAG	**167**	NR_033238.1

## Data Availability

The datasets generated for this study are available from the corresponding author upon request.
